# Clinal variation in investment into reproduction versus maintenance suggests a ‘pace-of-life’ syndrome in a widespread butterfly

**DOI:** 10.1007/s00442-020-04719-4

**Published:** 2020-07-27

**Authors:** Franziska Günter, Michaël Beaulieu, Kristin Franke, Nia Toshkova, Klaus Fischer

**Affiliations:** 1 Zoological Institute and Museum, Greifswald University, Soldmannstraße 14, 17489 Greifswald, Germany; 2 National Museum of Natural History at the Bulgarian Academy of Science, 1 Tsar Osvoboditel Blvd, 1000 Sofia, Bulgaria; 3 Present Address: Institute for Integrated Natural Sciences, University Koblenz-Landau, 56070 Koblenz, Germany

**Keywords:** Heat stress, Local adaptation, Molecular chaperones, Oxidative stress, *Pieris napi*

## Abstract

Extreme weather events such as heat waves are predicted to increase in the course of anthropogenic climate change. Widespread species are exposed to a variety of environmental conditions throughout their distribution range, often resulting in local adaptation. Consequently, populations from different regions may vary in their capacity to deal with challenging conditions such as thermal stress. In this study, we investigated clinal variation in body size, fecundity, and oxidative markers along a pan-European latitudinal gradient in the green-veined white butterfly *Pieris napi*, and additionally gene expression in German individuals. We exposed butterflies from replicated Italian, German, and Swedish populations to cold, control, or hot temperatures for 24 h. Under hot conditions, molecular chaperones were up-regulated, while oxidative damage remained unaffected and levels of the antioxidant glutathione (GSH) were reduced under cold and hot conditions. Thus, the short-term exposure to heat stress did not substantially affect oxidative balance. Moreover, we found decreased body size and fecundity in cooler compared with warmer regions. Interestingly, oxidative damage was lowest in Swedish animals exhibiting (1) high levels of GSH, (2) low early fecundity, and (3) low larval growth rates. These results suggest that Swedish butterflies have a slower life style and invest more strongly into maintenance, while those from warmer regions show the opposite pattern, which may reflect a ‘pace-of-life’ syndrome.

**Electronic supplementary material:**

The online version of this article (10.1007/s00442-020-04719-4) contains supplementary material, which is available to authorized users.

## Introduction

The significance of climate change, potentially affecting all organisms, is becoming more and more evident (Parmesan and Yohe 2003; Deutsch et al. 2008). Global surface temperatures rise, while precipitation patterns become more variable, which has already changed the distribution of many species (Diffenbaugh et al. 2005; Hansen et al. 2012). In particular, extreme weather events such as heat waves are predicted to increase, which may strongly affect biodiversity (Easterling et al. 2000; Meehl and Tebaldi 2004). To counteract the negative effects of changing environmental conditions, organisms may respond, in addition to shifting their range, through phenotypic plasticity and/or genetic adaption, thereby enhancing their performance under the novel environmental conditions which they encounter (Pigliucci 2001).

In widespread species, spatial variation in fitness-related traits often reflects differences in selective pressures, resulting in local adaptation (Ellers and Boggs 2002; Kawecki and Ebert 2004; Stillwell and Fox 2009). Clinal variation may indicate such local adaptation, as geographical clines are strongly related to environmental gradients, which may pose differential challenges to survival and reproduction. A well-known example of clinal variation is an increase in body size with increasing latitude (Bergmann size clines), the adaptive value of which though is not entirely clear with regard to ectotherms (Arnett and Gotelli 1999; Robinson and Partridge 2001; Chown and Klok 2003). Indeed, short season length and low ambient temperature at higher latitudes may select for fast growth, which may result in converse Bergmann clines (Van Doorslaer and Stoks 2005). In the face of anthropogenic climate change, investigating widespread species showing local adaptation is important, as such species may respond differently across their distribution range to ongoing environmental change depending on their genetically based plastic capacities (Addo-Bediako et al. 2000; Hoffmann and Rieseberg 2008; Kapun et al. 2016).

Plastic responses to environmental change entail a large number of relatively fast physiological mechanisms. In response to thermally challenging conditions, two mechanisms seem to be of particular importance, antioxidant defence mechanisms and the heat shock response (Sørensen et al. 2003; Zhang et al. 2015; Franke et al. 2019). High temperatures generally increase the production of reactive oxygen species (ROS), a by-product of the aerobic metabolism, which act on organic substrates and may induce oxidative damage. This can, for instance, be measured with markers of lipid peroxidation such as malondialdehyde (MDA; Beaulieu et al. 2015; Zhang et al. 2015). To prevent oxidative damage induced by high temperatures, organisms may upregulate various antioxidant compounds, including the non-enzymatic antioxidant glutathione (GSH; Lalouette et al. 2011; Ju et al. 2014). In parallel, organisms may upregulate heat shock proteins (HSP), which act as molecular chaperones that help organisms to cope with a wide range of stressors (Sørensen et al. 2003). Such defence mechanisms are expected to be costly and may compromise other fitness components such as reproduction (Alonso-Alvarez et al. 2004; Beaulieu et al. 2015). Mechanisms against heat exposure likely vary across the distribution range of widespread species because of local adaptation, which may disadvantage some populations relative to others in the face of climate warming.

We here investigate variation in body size, fecundity, and physiological defence mechanisms in relation to short-term thermal stress along a latitudinal gradient in the green-veined white butterfly *Pieris napi*. This species has a wide distribution range and is known to show local adaptation (Günter et al. 2019, 2020). Specifically, we focus on geographic variation in physiological responses to short-term temperature stress, including a simulated hot summer day which will increase in frequency with climate change. Towards this end, we exposed butterflies from replicated Italian, German, and Swedish populations for 24 h to cold, control, or hot conditions. Physiological responses were scored by measuring MDA and GSH levels as well as gene expression. Because of potential trade-offs between physiological defence mechanisms and other fitness components, we also examined body size and fecundity in parallel to defence mechanisms. Based on a known converse Bergmann cline (Blanckenhorn et al. 2006; Nygren et al. 2008) in body size in *P. napi* (Petersen 1947; Nylin and Svärd 1991; Günter et al. 2020), we predicted smaller individuals at higher latitude, which may be associated with slower growth and decreased fecundity (Honěk 1993; Blanckenhorn 2000). A reduced fecundity may potentially allow for increased investment into defence mechanisms in individuals from high latitude in response to thermal stress. Accordingly, we predicted an up-regulation of antioxidant defence mechanisms to prevent oxidative damage. Note though that fecundity can be affected by many factors including direct selection, for instance in relation to the time available for egg-laying (Springer and Boggs 1986). This evidently complicates predictions concerning fecundity and associated trade-offs.

## Materials and methods

### Study organism and experimental populations

The green-veined white butterfly *Pieris napi* L. is a widespread temperate-zone butterfly, occurring throughout Europe and the temperate zone of Asia (Ebert and Rennwald 1993). It is one of the most common butterflies in Europe. Nevertheless, it is predicted to suffer from anthropogenic climate change because of its association with moist habitats (Fox et al. 2015). Larvae feed on a variety of Brassicaceae, with *Alliaria petiolata* Cavara & Grande being the most important one. Brassicaceae are also the preferred nectar plants. The species overwinters in the pupal stage (Wiklund et al. 1991) and has typically one to four generations per year (Müller and Kautz 1938; Tolman and Lewington 2008). *P. napi* is polyandrous with males transferring large nuptial gifts to their female partners (Stjernholm and Karlsson 2000; Bergström and Wiklund 2002). Accordingly, males are larger than females (Wiklund and Kaitala 1995).

**Fig. 1:**
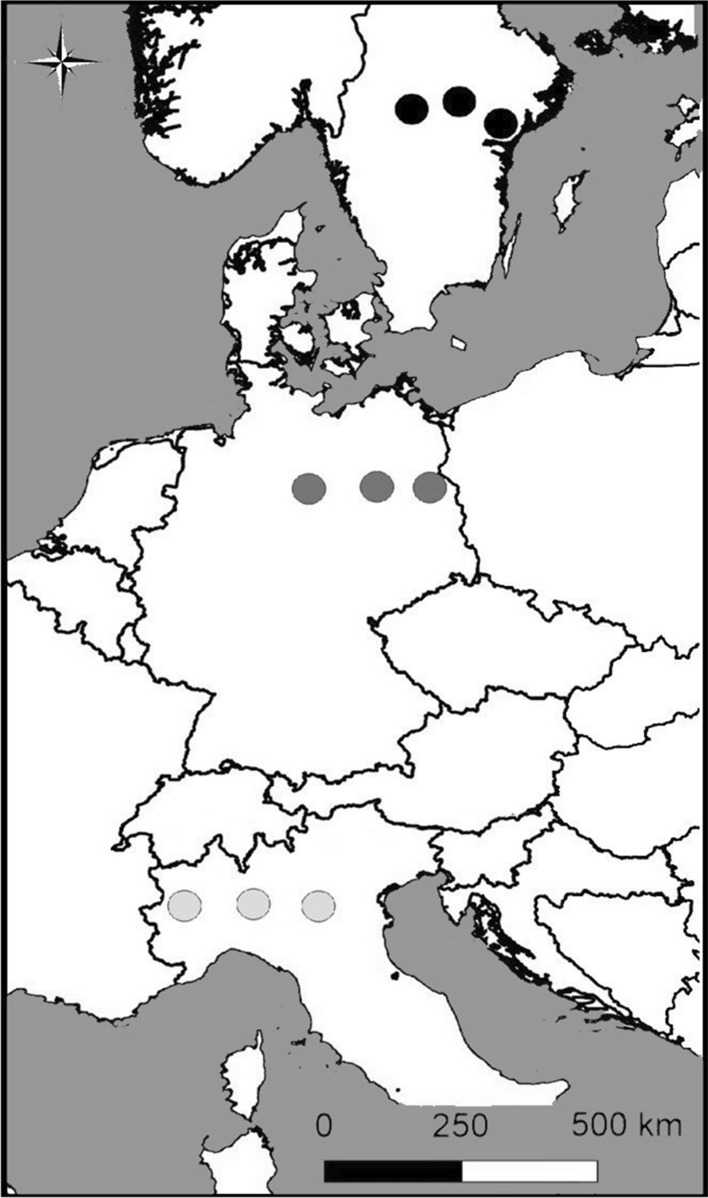
Sampling locations of *Pieris napi* individuals used in the present study. Populations were collected in Italy (light grey circles), Germany (dark grey circles), and Sweden (black circles); modified after (Günter et al. 2019)

For this study, we collected freshly enclosed spring generation females along a latitudinal gradient from northern Italy to Sweden. We sampled three replicate populations each in northern Italy (I: Torino 45.11° N/7.48° E, Pavia 45.21° N/9.27° E, Mantova 45.21° N/10.75° E), northern Germany (G: Wahrenholz 52.64° N/10.61° E, Rathenow 52.65°N/12.44° E, Strausberg 52.60° N/13.86° E), and South Sweden (S: Örebro 59.29° N/15.01° E, Eskilstuna 59.36° N/16.54° E, Stockholm 58.95° N/17.58° E; Fig. 1). Mean annual temperatures follow a latitudinal gradient (Italy: 13 °C, Germany, 9 °C, Sweden: 6 °C), while mean annual precipitation is higher in Italy (865 mm) than in Germany (570 mm) and Sweden (578 mm; Günter et al. 2019). Likewise, the mean temperature during the flight period (May–September; Italy: 20.6 °C, Germany: 15.8 °C, Sweden: 14.2 °C) as well as the mean maximum temperature (Italy: 25.9 °C, Germany: 23.4 °C, Sweden: 18.8 °C) follow a latitudinal gradient. Special Report on Extreme Events (IPCC 2012) concludes that there have been major increases in the frequency of warm temperature extremes in Europe, with high confidence for the Mediterranean region. Over North and Central Europe, there is medium confidence in an increase in heat-wave intensity and frequency.

The minimal straight distance between two populations was 73 km, the total latitudinal gradient spanned ca. 1660 km. We collected a total of 74 females from Italy, 94 from Germany, and 76 from Sweden between 19 April and 14 June 2016. All females were subsequently transferred to the University of Greifswald for egg-laying.

We have used this latitudinal gradient and the according populations before to investigate (1) phenotypic variation in field-collected males (Günter et al. 2019) and (2) genetic variation and developmental plasticity (Günter et al. 2020). We found decreased body size, increased wing melanisation, and reduced yellow reflectance with increasing latitude (and altitude in additional populations) in field-collected males (Günter et al. 2019). Using a common garden design, we found that individuals from cooler environments were less heat-tolerant, had a longer development time but were, nevertheless, smaller, and had more strongly melanised wings (Günter et al. 2020). In addition, we showed that a higher developmental temperature speeded up development, reduced body size, potential metabolic activity, and wing melanisation but increased heat tolerance.

### Experimental design

Field-caught females were kept individually in translucent 1-l plastic pots covered with gauze, which were placed into a climate chamber set at a photoperiod of L18:D6, a constant temperature of 25 °C, and 65% relative humidity. Females were fed ad libitum with water, a 10% sugar solution, and additionally flowers (e.g. *Sambucus nigra*, *Taraxacum* spec., *Senecio* spec.). For oviposition, they were provided daily with a fresh cutting of *A. petiolata*. The resulting eggs were collected daily and transferred, separated by females, to elongated, sleeve-like gauze cages. Resulting larvae were fed with fresh cuttings of *A. petiolata* and young rape plants (*Brassica napus*). All plants were replaced as necessary and the density per cage was limited to a maximum of 40 larvae. Rearing took place in climate chambers under the same conditions as for egg-laying. One day after pupation, resulting pupae were transferred to 500 ml plastic boxes containing moist tissue. On the day of adult eclosion, butterflies were mated within populations, though not allowing siblings to mate. After mating, females were transferred to translucent 1-L plastic pots covered with gauze and containing nectar plants, a leaf of *A. petiolata* for egg-laying, 10% sugar solution, and water ad libitum. They were maintained under such conditions for 3 days to lay eggs. Eggs were collected and counted daily. We scored early fecundity as a proxy for reproductive investment to avoid mortality before allocation to treatments (note that animals need to be frozen alive for scoring physiological traits). Butterflies typically lay the majority of eggs early within the oviposition period, and early fecundity correlates strongly with lifetime fecundity (Brakefield et al. 2001; Fischer and Fiedler 2001; Kehl et al. 2015). Furthermore, for scoring trade-offs with short-time physiological responses, current reproductive investment seems more relevant than lifetime investment.

**Fig. 2:**
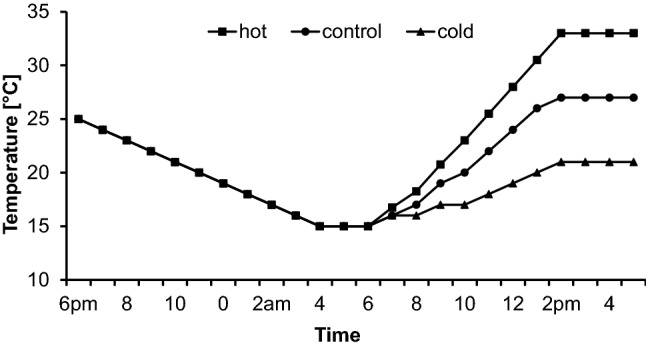
Temperature profiles of the 3-day cycle treatments. Butterflies were allocated to treatments at 6 p.m. and frozen 24 h later

Afterwards, females were evenly divided among three thermal treatments characterized by cold, control, and hot conditions (Fig. 2). The daily temperature cycles used were meant to reflect a cool, average, or hot summer day. All treatments started at 25 °C, with a progressive reduction to 15 °C to simulate evening/night conditions. Thereafter, temperatures progressively increased to 21 °C (cold), 27 °C (average) or 33 °C (hot). We used short-time exposure to different thermal regimes as according physiological responses can assumed to be very fast. For example, heat shock proteins were found to be up-regulated within hours after 1-h exposure only (Karl et al. 2012). Per treatment, a single climate cabinet was used with identical settings except for thermal profiles (Sanyo MIR-553; Bad Nenndorf, Germany). Males, in contrast, were allocated to treatments immediately after mating. Butterflies were kept for 24 h at the respective thermal treatment. Afterwards, they were frozen at − 80 °C for later analyses. Offspring sample sizes were 0, 47, and 13 for Swedish, 60, 77, and 57 for German, and 59, 41, and 39 for Italian populations.

The head, wings, and legs were removed from frozen butterflies, and the thorax and abdomen were separated before being weighed (KERN ABJ-120-4M). For each individual (except those used for transcriptome analyses, see below), we measured thorax mass, abdomen mass, and two physiological parameters related to oxidative stress: a marker of antioxidant defence, glutathione (GSH, a non-enzymatic antioxidant), and a marker of oxidative damage, malondialdehyde (MDA, a marker of lipid peroxidation). Glutathione is a central endogenous antioxidant protecting cells against the action of ROS by scavenging them directly or through enzymatic reactions (Aquilano et al. 2014), while MDA is a marker of oxidative damage on lipids, the class of biomolecules most affected by the action of ROS (Del Rio et al. 2005). For measuring GSH and MDA, we used the methods detailed in Günter et al. (2020). In short, we homogenized thoraces and abdomen with a TissueLyser (QIAGEN, The Netherlands) through 1 min and then cooled down for 1 min on dry ice. This procedure was repeated three times. The resulting homogenates were centrifuged at 16,249 g for 30 min at 4 °C. Then, the supernatants were transferred to new tubes and centrifuged once again for 15 min. The resulting supernatants were used to analyse total protein concentration, MDA, and GSH. Total protein concentration was determined at 595 nm using a microplate reader (Epoch 2, BIO-TEK Instruments) according to the Bradford method.

GSH levels were assessed with a spectrophotometric method that involves oxidation of GSH by 2-nitrobenzoic acid to the yellow derivative 5′-thio-2-nitrobenzoic acid (TNB). A GSH stock solution was prepared from 100 mM GSH and diluted to a standard series. Per 1 mg protein sample, sulfosalicylic acid (end concentration 4%) was added in 150 µl volume and incubated overnight at 4 °C. Afterwards, samples were centrifuged at 16,249*g* for 30 min at 4 °C, and neutralized using 1 M NaOH until reaching pH 7. Each probe was diluted using a factor of 1:2 and 1:4. Standards, samples, and dilutions were transferred to a microplate. Then, 40 µl 1.5 mM 2-nitrobenzoic acid was added to each well to start the assay. Absorbance was measured at 412 nm with 30 kinetic cycles. MDA concentrations were measured with a commercially available Microplate Assay Kit (CAK1011, Cohesion Bioscience) by reading the maximum absorbance at 532 nm and 37 °C (Meng et al. 2009). GSH and MDA values were corrected by protein content.

### Transcriptome analyses

We used transcriptome analyses to prove that the treatments used actually imposed thermal stress and to investigate which physiological pathways are activated by butterflies exposed to hot conditions. For logistic reasons, analyses were restricted to German individuals previously exposed to control or hot conditions. We randomly selected two pairs of full-sib sisters per population (*n* = 12 individuals), with one sibling being exposed to control and the other to hot conditions. Whole thoraces and abdomens were used for RNA extraction. RNA isolation was carried out using TRIZOL (Invitrogen) according to the manufacturer’s instructions, using 1 mL of TRIZOL per individual. Animal tissue was disrupted using a tissue lyser (Qiagen 20.747.0001; bead beating with 5 mm steel beads) for 1 min at 30 Hz. For RNA purification, the RNeasy Mini kit (Qiagen 74106) was used. Construction of the RNA library and transcriptomic sequencing were performed by LGC Genomics GmbH (Berlin, Germany). For details, see Supplementary Material S1. Changes in the level of gene expression are presented as Log2-fold change (Log2FC) values. They show whether genes are up- or down-regulated and how strongly so. A value < 1 reflects a down-regulation, while a value > 1 reflects an up-regulation.

### Statistical analyses

Data on egg number, morphology, and physiology were analysed using general linear mixed models (GLMMs) with country (Italy, Germany, Sweden), sex, treatment (cool, benign, hot), and all resulting interactions as fixed factors, and replicate population as random effect nested within country. Egg numbers were analysed only for females irrespective of thermal treatment (as eggs were collected before allocation to treatments). MDA, GSH, and egg number were square root-transformed prior to analyses to meet GLMM requirements. Models were constructed by stepwise backward removal of non-significant interactions. For egg number, we additionally performed covariance analyses by adding thorax and abdomen mass or MDA and GSH as covariates. Throughout the text, means are given ± 1 SE. All statistical tests were performed with Statistica 12.0 (Tulsa, StatSoft, OK).

## Results

### Egg number, morphology, and physiology

**Fig. 3:**
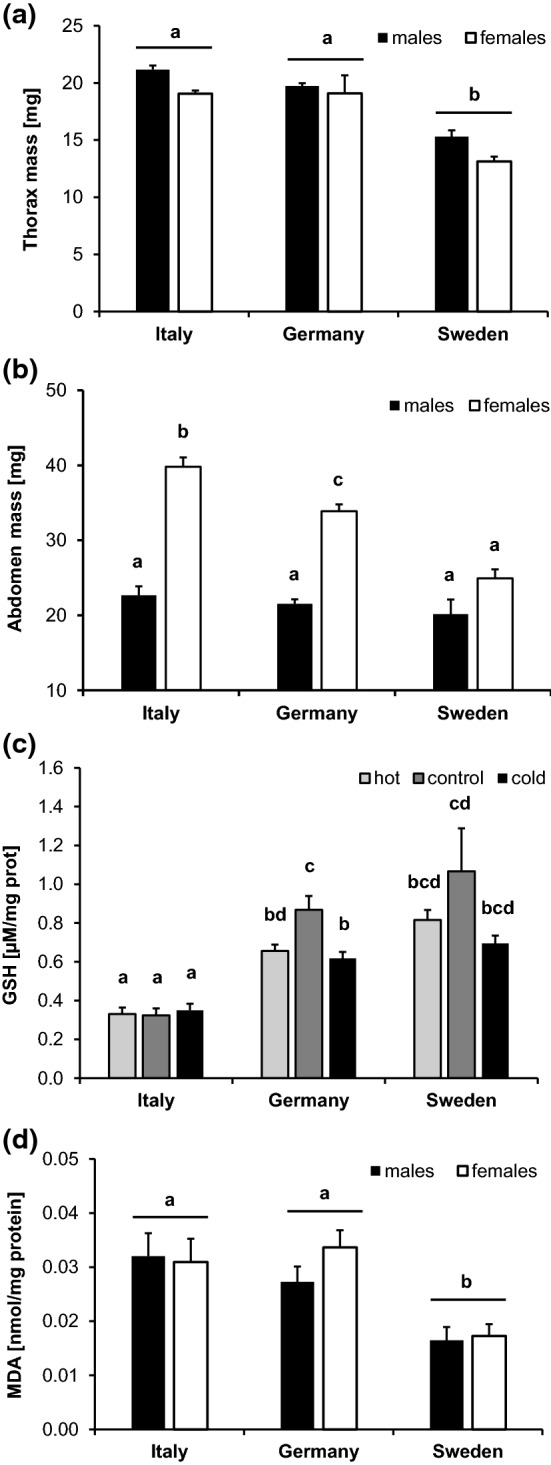
Means ± SE for thorax mass (**a**), abdomen mass (**b**), GSH (**c**), and MDA (**d**) for *Pieris napi* in relation to country of origin and sex (**a**, **b**, **d**) or treatment (**c**). Different letters above bars show significant differences among groups (Tukey HSD after GLMM)

The number of eggs collected over a 3-day period following mating was significantly higher in Italian (57.1 ± 5.3 eggs) than Swedish females (15.8 ± 5.2), with German ones showing intermediate values (43.7 ± 3.8; Tukey HSD after GLMM: I ≥ G ≥ S; *F*_2,9_ = 4.9, *P* = 0.037; population *F*_5,151_ = 0.9, *P* = 0.458). Thorax mass, abdomen mass, MDA, or GSH were not related to egg number significantly when added as covariates (all *P* values > 0.10). Origin significantly affected thorax mass, abdomen mass, MDA, and GSH, while significant population differences were found for abdomen mass and GSH only (Table 1). Thorax and abdomen mass as well as MDA decreased from South (Italy) to North (Sweden), while the opposite pattern was observed for GSH (Fig. 3). For abdomen mass, the latitudinal gradient was restricted to females (significant country-by-sex interaction; Fig. 3b). Thermal treatment had a significant effect on GSH only, with values being higher under control than cold or hot conditions (control: 0.667 ± 0.052 µM/mg protein > cold: 0.542 ± 0.025 = hot: 0.564 ± 0.027; Tukey HSD after GLMM). This pattern was, however, only significant in German butterflies, although there was a similar trend in Swedish butterflies (significant country by treatment interaction; Fig. 3c).

**Table 1:** Results of general linear mixed models testing for the effects of country (fixed factor), population (random, nested within country), sex and temperature treatment (both fixed) on thorax mass, abdomen mass, glutathione (GSH), and malondialdehyde (MDA) in *Pieris napi* from a latitudinal gradient

Thorax mass	MS	DF	*F*	*P*
Country	5.8 × 10^–04^	2, 15	21.1	**< 0.0001**
Population [country]	2.0 × 10^–05^	5, 381	0.3	0.8907
Sex	1.9 × 10^–04^	1, 381	3.3	0.0706
Treatment	2.1 × 10^–06^	2, 381	0.0	0.9651
Error	5.9 × 10^–05^	381		

Abdomen mass	MS	DF	*F*	*P*
Country	1.0 × 10^–03^	2, 6	5.5	**0.0417**
Population [country]	1.7 × 10^–04^	5, 341	2.4	**0.0376**
Sex	9.7 × 10^–03^	1, 341	112.0	**< 0.0001**
Treatment	1.3 × 10^–04^	2, 341	1.7	0.1817
Country × sex	7.8 × 10^–04^	2, 341	7.0	**0.0011**
Error	7.1 × 10^–05^	341		

GSH	MS	DF	*F*	*P*
Country	3.316	2, 6	46.0	**0.0002**
Population [country]	0.082	5, 343	2.3	**0.0441**
Sex	0.039	1, 343	1.1	0.2941
Treatment	0.172	2, 343	4.8	**0.0084**
Country × treatment	0.103	4, 343	2.9	**0.0219**
Error	0.036	343		

MDA	MS	DF	*F*	*P*
Country	0.022	2, 11	5.9	**0.0179**
Population [country]	0.003	5, 377	0.5	0.7639
Sex	0.003	1, 377	0.5	0.4750
Treatment	0.005	2, 377	0.7	0.4791
Error	0.006	377		

Models were constructed by stepwise backwards elimination of non-significant interactions. Significant *P* values are given in bold

### Gene expression

**Fig. 4:**
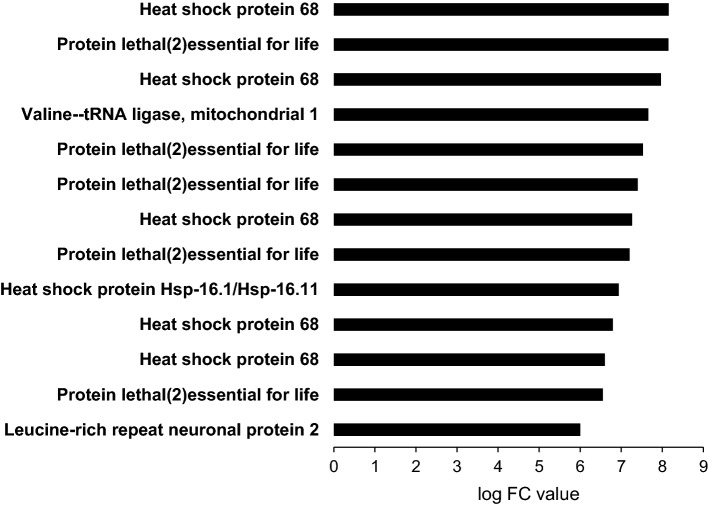
Overview of the genes being most strongly up-regulated under hot compared to control temperatures in *Pieris napi* from Germany. Only genes with log2FC values > 6 are shown

In German female butterflies, we found 25 annotated transcripts that were significantly up-regulated under hot compared with control temperatures (Supplementary Material S2). Log2FC values for these transcripts ranged between 3.1 and 8.2. As several transcripts belonged to the same gene, we identified only 15 according genes (Table 2). These genes were mainly associated with cellular processes and signalling (11). Two genes were related to information storage and processing, while the function of the two remaining genes was unknown. Nine out of the fifteen genes were molecular chaperones, two were involved in ‘translation, ribosomal structure and biogenesis’, and two in ‘signal transduction mechanisms’ (two had unknown functions). The genes most strongly up-regulated were heat shock protein 68, lethal (2) essential for life, and Valyl-tRNA synthetase (Fig. 4).

**Table 2:** Functional annotation of transcripts up-regulated in *Pieris napi* from Germany at the higher temperature, including protein and gene name, description, molecular main, and subrole

gi No.	Protein name	Gene	Description	Main role	Subrole
17738165	Heat shock protein 68	Hsp68	Molecular chaperones HSP70/HSC70, HSP70 superfamily	Cellular	Chaperones
1170372	Heat shock protein 70 A1	Hsp70A1	Molecular chaperones HSP70/HSC70, HSP70 superfamily	Cellular	Chaperones
665390828	Heat shock 70 kDa protein cognate 3	Hsc70-3	Molecular chaperones GRP78/BiP/KAR2, HSP70 superfamily	Cellular	Chaperones
3096951	Heat shock protein 90	Hsp90	Molecular chaperone, HSP90 family	Cellular	Chaperones
54642233	Heat shock protein 83	Hsp83	Molecular chaperone, HSP90 family	Cellular	Chaperones
442624575	Protein lethal (2) essential for life	l(2)efl	Alpha crystallins	Cellular	Chaperones
17562026	Heat shock protein 16.1/16.11	Hsp-16.1	Alpha crystallins	Cellular	Chaperones
81909571	Heat shock protein beta-6	Hspb6	Alpha crystallins	Cellular	Chaperones
197102236	DnaJ homolog sub-family A member 1	DNAJA1	Molecular chaperone, DnaJ superfamily	Cellular	Chaperones
21542452	Valine-tRNA ligase, mitochondrial 1	TWN2	Valyl-tRNA synthetase	Information	Translation
74870264	Putative tRNA pseudo-uridine synthase	Pus10	Predicted pseudouridylate synthase	Information	Translation
21706506	Leucine-rich repeat neuronal protein 2	LRRN2	Membrane glycol-protein LIG-1	Cellular	Transduction
75273276	Probable protein phosphatase 2C 38	PP2C38	Protein phosphatase 2C/pyruvate dehydrogenase phosphatase	Cellular	Transduction
130398	Retrovirus-related Pol polyprotein	Pol	Unknown	–	–
1326016	Transposon Ty3-I Gag-Pol polyprotein	TY3B-I	Unknown	–	–

Main roles: cellular—cellular processes and signalling; information—information storage and processing. Subroles: chaperones—posttranslational modification, protein turnover, chaperones; translation—translation, ribosomal structure and biogenesis; transduction—signal transduction mechanisms

## Discussion

We found clinal variation in body mass, which decreased with increasing latitude. Thus, individuals from warmer (Italy) rather than cooler regions (Sweden) were larger, confirming the known converse Bergmann’s rule in *P. napi* (Petersen 1947; Nylin and Svärd 1991). We thus assume that warmer conditions are more beneficial for the development of *P. napi* allowing for larger body size (David and Gardiner 1962; Angilletta and Dunham 2003), causing this genetically based cline. Several examples for converse Bergmann size clines have already been reported (Blanckenhorn and Demont 2004; Blanckenhorn et al. 2006), for instance based on short season length and low ambient temperatures at higher latitudes constraining growth opportunities (Van Doorslaer and Stoks 2005).

Females had much heavier abdomen than males, which is likely related to egg production. As the males and females used here were mated, the sexual difference in abdomen mass may also arise from the large spermatophores that males transfer to females in *P. napi* (up to 15% of the males’ initial body mass; Svärd and Wiklund 1989; Wiklund and Kaitala 1995). In general, high abdomen mass in female insects is related to reproductive investment and hence high fecundity (Tammaru et al. 1996; Berwaerts et al. 2002). Accordingly, we found that early fecundity showed the same pattern than abdomen mass, with more eggs being laid by heavier Italian than by lighter Swedish females. This suggests that higher female abdomen mass enables high early fecundity in southern populations. Perhaps, fast reproduction is advantageous in warmer climates, as large egg numbers can be laid during a single warm day allowing for long activity.

Interestingly, MDA, which is a degradation product of polyunsaturated fatty acids and thus a marker for lipid peroxidation through ROS (Liu et al. 1997; Janssens et al. 2017), was lower in Swedish than in Italian or German individuals. The reduced oxidative damage may be related to relatively high levels of antioxidants (here: GSH) in Swedish individuals. The antioxidant GSH is known to efficiently remove ROS (Hayes and McLellan 1999; Kregel and Zhang 2007). This suggests that butterflies from higher latitudes invest more strongly into self-maintenance mechanisms than butterflies from lower latitudes. This higher investment may in turn be, at least partly, enabled by the lower reproductive investment and the overall slower lifestyle (Günter et al. 2020) of Swedish animals. Indeed, variation in antioxidative defences has been found in relation to reproduction, larval developmental time, growth rate, wing melanisation rate, and heat resistance (Günter et al. 2019, 2020). However, we did not find a direct link between fecundity and oxidative markers. Thus, despite indications for a micro-evolutionary trade-off, a similar physiological trade-off could not be confirmed. We, therefore, have to reject our hypothesis that females showing a high reproductive investment inevitably suffer more from oxidative stress. This negative finding may result from (1) measuring only two markers related to oxidative stress, (2) too short exposure to different temperature profiles, (3) using temperatures that were not extreme enough (despite a strong up-regulation of molecular chaperones, see below), or (4) not enough time for physiological responses to occur. Regarding the latter, very little is known on the temporal dynamics of the up-regulation of antioxidant defences and the occurrence of oxidative damage (Schlorff et al. 1999). Anyway, short-time exposure to mildly stressful conditions does not seem to necessarily result in a trade-off between maintenance and reproduction. In line with this conclusion, temperature treatment did not affect MDA. GSH, in contrast, was significantly affected by temperature. Interestingly, highest levels were found under control conditions, which may indicate that hot as well as cold conditions may interfere with GSH. The decrease in GSH under hot conditions is likely due to a higher production of ROS under such conditions and by the resulting higher oxidation of GSH into GSSG (Hayes and McLellan 1999; Kregel and Zhang 2007).

The transcriptome analyses also showed no pronounced variation among temperature treatments in markers related to oxidative stress. However, several molecular chaperones, including heat shock proteins and alpha crystallins, were up-regulated under hot conditions, which is a well-known response to heat stress across various taxa (Sørensen et al. 2001; Karl et al. 2009). These proteins participate in folding and unfolding of proteins and help maintaining their secondary structure under extreme environmental conditions (Parsell and Lindquist 1993). Our results on gene expression confirm that the treatments used were appropriate for elucidating short-term physiological responses, at least with regard to the heat shock response in German females.

## Conclusions

Our study revealed clinal variation in body mass and fecundity (both being higher in warmer regions), and oxidative defence mechanisms. Regarding the latter, oxidative damage was lowest in Swedish animals, having at the same time the highest levels of the antioxidant GSH. This may reflect geographical variation in life styles and accordingly antioxidant defence mechanisms. Swedish individuals showed the lowest early fecundity in the current study, and are also known to have the lowest growth rates (Günter et al. 2020). Thus, Swedish individuals seem to have a slower life style and to invest more strongly into maintenance, while Italian individuals show the opposite pattern, which may reflect a ‘pace-of-life’ syndrome (Montiglio et al. 2018). Note though that we could not show a direct link between fecundity and physiological parameters. Exposing individuals for 24 h to different temperature treatments caused an increased expression of molecular chaperones, but did not affect oxidative damage, while levels of the antioxidant GSH were reduced under cold and hot conditions. We conclude that short-term exposure to heat stress does not substantially affect oxidative balance. The effects which longer exposure times though may have on oxidative stress and its relations to reproduction and maintenance deserves further attention.

## Electronic supplementary material

Below is the link to the electronic supplementary material.

Supplementary file1 (DOCX 15 kb)
