# Supplementary material for: Clinal variation in investment into reproduction versus maintenance suggests a ‘pace-of-life’ syndrome in a widespread butterfly
Source: Oecologia. 2020 Jul 27;193(4):1011–20. doi: 10.1007/s00442-020-04719-4 (PMC7458933; doi:10.1007/s00442-020-04719-4)
Supplement: Supplementary file 1 — Supplementary file1 (DOCX 15 kb) [file 442_2020_4719_MOESM1_ESM.docx]

**Supplementary Material S1**

**RNA isolation and purification**

Construction of the RNA library and transcriptomic sequencing were performed by LGC Genomics GmbH (Berlin, Germany). Whole thoraces and abdomens were used for RNA extraction (n = 12). We selected 6 butterfly families that means the offspring of 6 single-mated females. RNA isolation was carried out using TRIZOL (Invitrogen) including bead beating (5mm stainless steel; 1min 30 Hz in Tissue Lyser (QIAGEN)) followed by a clean-up of the supernatant using Qiagen RNeasy Mini Kit.

**Sequencing, transcriptome assembly, and expression analysis**

First, mRNA was isolated via oligo dT binding and using the NEBNext Poly(A) Magnetic Isolation Module (New England Biolabs) according to the manual. First strand cDNA was synthesized using a NEBNext RNA First Strand Synthesis Module (New England Biolabs) according to the manual. Subsequently, second strand cDNA synthesis was performed using NEBNext RNA Second Strand Synthesis Module (New England Biolabs) according to the manual. The library fragments were purified with MinElute Columns (Qiagen), eluted in 20μl EB buffer. Concentration measurement (Qubit) showed a cDNA yield of about 2ng/μl for all samples. The Encore Rapid DR Multiplex system (Nugen) was used for library preparation according to the manual. Libraries were amplified in a volume of 100μl for 15 cycles using MyTaq (Bioline) and standard Illumina primers. The size selection was done on a preparative Agarose Gel selecting fragments between 300 and 500bp

We demultiplexed all libraries for each sequencing lane with using the Illumina bcl2fastq 2.17.1.14 software. In total, we obtained 150 bp paired-end reads (Illumina NextSeq 500 V2), with 400 million read pairs.

In the next step we clipped off sequencing adapter remnants from all raw reads (reads with final length < 20 bases were discarded). Quality trimming of adapter-clipped reads were executed by using Trinity 2.2.0 grouped digital normalization (<http://trinityrnaseq.sourceforge.net/>). rRNA sequences from adapter clipped reads were filtered by using RiboPicker 0.4.3 (http://ribopicker.sourceforge.net/). Given that a reference genome of *P. napi* was not available, we performed a de novo assembly. The digitally normalized Illumina reads were de novo assembled with Trinity 2.2.0. Peptide identification was done with TransDecoder rel16JAN2014 (<http://transdecoder.sourceforge.net/>). The draft functional annotation of transcontigs and predicted peptides was done with Trinotate r20131110 (<http://trinotate.sourceforge.net/>).

**Differential expression analysis**

To identify differential expression of assembled transcripts, we aligned the them against assembled reference with STAR 2.4. (<https://github.com/alexdobin/STAR/releases>). The post-alignment filtering of reads was aligned to rRNA or tRNA regions. We counted the aligned reads with htseq-count (<http://www-huber.embl.de/users/anders/HTSeq/>). The differential expression analysis was done by edgeR 3.2.3 (http://www.bioconductor.org/packages/release/bioc/html/edgeR.html) and DESeq 1.12.0 (<http://bioconductor.org/packages/release/bioc/html/DESeq.html>). In the last step we combined and filtered the differential expression analysis. Significance was determined using a false discovery rate (FDR) threshold of 5%. The assembled transcript clusters were annotated against eukaryotic orthologous groups (KOG; RPS-BLAST algorithms) using the program Prophane and NCBI gi numbers. The COG/KOG system distinguishes among two protein main roles and 3 sub-roles.
